# Nasal Fistula Associated With Dental Infection Following Severe Tooth Injury: A Case Study

**DOI:** 10.7759/cureus.111862

**Published:** 2026-07-01

**Authors:** Manisha Verma, Mohammad Abdurrahman Khan, Pratibha Dwivedi

**Affiliations:** 1 Department of Dentistry, Hind Institute of Medical Sciences, Lucknow, IND; 2 Department of Forensic Medicine and Toxicology, Hind Institute of Medical Sciences, Lucknow, IND; 3 Department of Anatomy, Hind Institute of Medical Sciences, Lucknow, IND

**Keywords:** extraoral sinus tract, nasal fistula, odontogenic infection, peri-apical lesion, surgical curettage

## Abstract

Dental pulp infection is predominantly initiated by anaerobic, gram-negative bacterial flora introduced through dental caries, operative dental procedures, or severe tooth injury, which can lead to pulp necrosis. A 39-year-old man developed an extra-nasal sinus tract on the right side of the nostril following trauma to the right maxillary central incisor. He presented with a chief complaint of a painful mass inside the right nostril after the right maxillary central incisor was traumatized in a fall down the stairs. A diagnosis of a periapical abscess with a sinus tract on the right side of the nostril was made. The patient was managed with endodontic treatment of the non-vital maxillary central incisor followed by debridement of the periapical lesion, resulting in complete resolution of the lesion and nasal swelling. Clinicians should be aware of both odontogenic and non-odontogenic causes of lesions associated with sinus pathologies and should carefully evaluate the pathways of infection through thorough clinical and radiographic examination.

## Introduction

Sinus tracts often present a diagnostic challenge. Extraoral sinus tracts are commonly associated with odontogenic infections, whereas intraoral sinus tracts are typically of odontogenic origin [1]. The occurrence of a nasal sinus tract of dental origin is very rare [2]. Pulp necrosis and periapical infection may result in sinus tract formation [1-3]. Dental pulp infection is predominantly caused by anaerobic gram-negative bacterial flora introduced through dental caries, operative dental procedures, or severe tooth injury, leading to pulp necrosis [4]. Local immune responses initiated by pulpal infection are often prolonged beyond the elimination of the invading microorganisms [5]. Consequently, pulpal necrosis can stimulate a secondary immune response in the periapical region [6]. Depending on their cellular composition and structural organization, these lesions are classified as abscesses, granulomas, or periapical cysts. Suppuration associated with a periapical abscess may lead to spontaneous drainage of pus, resulting in the formation of a drainage tract, known as a fistula or sinus tract [1,6]. Generally, when pus accumulates beneath the mucosa, it produces localized swelling that eventually ruptures, allowing pus to drain into the oral cavity. The intraoral opening is usually visible on the attached buccal gingiva or in the vestibule. Less commonly, fistulas may occur in the nasal cavity, present as cutaneous sinus tracts, or extend into the maxillary sinus [2,7,8]. A sinus tract may also present extraorally on the face or neck. If acute periapical inflammation persists, it may progress to chronic inflammation, leading to sinus tract formation with persistent pain and continuous drainage. These symptoms typically resolve only after appropriate endodontic treatment or extraction of the affected tooth if it has a hopeless prognosis [6]. In the present case, the patient presented with an unusual swelling inside the right nostril, accompanied by continuous purulent drainage, severe inflammation, pain, and redness around the nostril. The condition was successfully managed with conventional root canal treatment followed by periapical surgical curettage.

## Case presentation

A 39-year-old male patient presented to the dental department of our tertiary care center with a chief complaint of a painful mass inside the right nostril that had been present for the past 4-5 months. The patient also reported a history of trauma due to a fall down the stairs, during which his maxillary anterior teeth were injured and left untreated for several years. He had taken medications prescribed by a local dentist; however, there was no improvement in the lesion or symptoms. Clinical examination revealed black discoloration of the right maxillary central incisor due to pulp necrosis (Figure 1).

**Figure 1:**
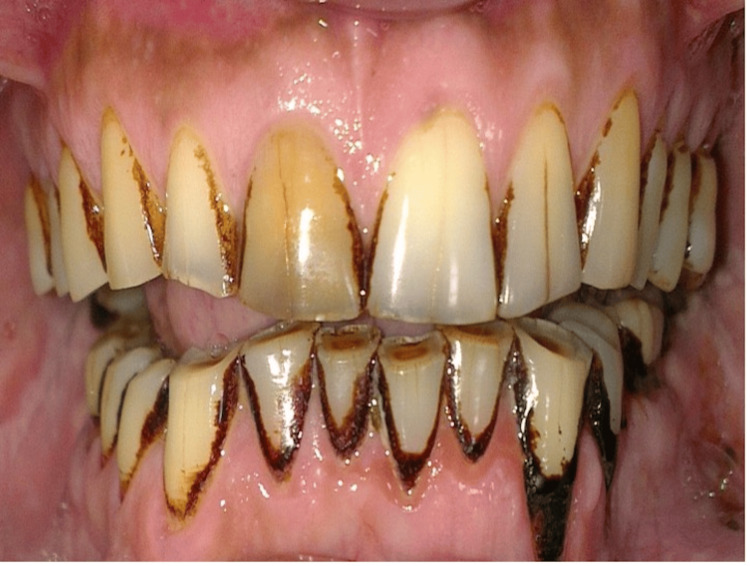
Discolored right maxillary central incisor following trauma

He also complained of intermittent discharge from the nasal sinus tract, which was associated with significant pain. The patient had a 20-year history of tobacco chewing. Severe tooth attrition, cervical abrasion, and enamel microcracks were also noted. The patient had no significant medical history.

Radiographic examination, including an intraoral periapical radiograph (IOPA), revealed a periapical lesion (Figure 2) at the root apex of the right maxillary central incisor. The tooth had a widened root canal due to previous trauma and was non-vital and discolored. The sinus tract on the right side of the nostril was explored by inserting a gutta-percha cone toward the apex of the right maxillary central incisor, confirming the presence of a periapical lesion of odontogenic origin (Figure 3 and Figure 4).

**Figure 2:**
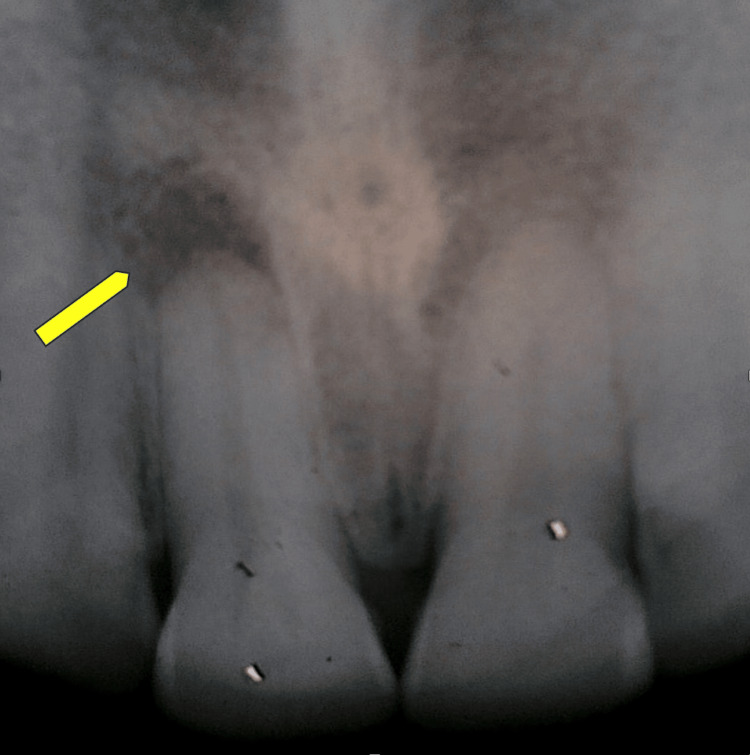
Intraoral periapical (IOPA) radiograph showing a periapical lesion associated with the infected right maxillary central incisor (yellow arrow)

**Figure 3:**
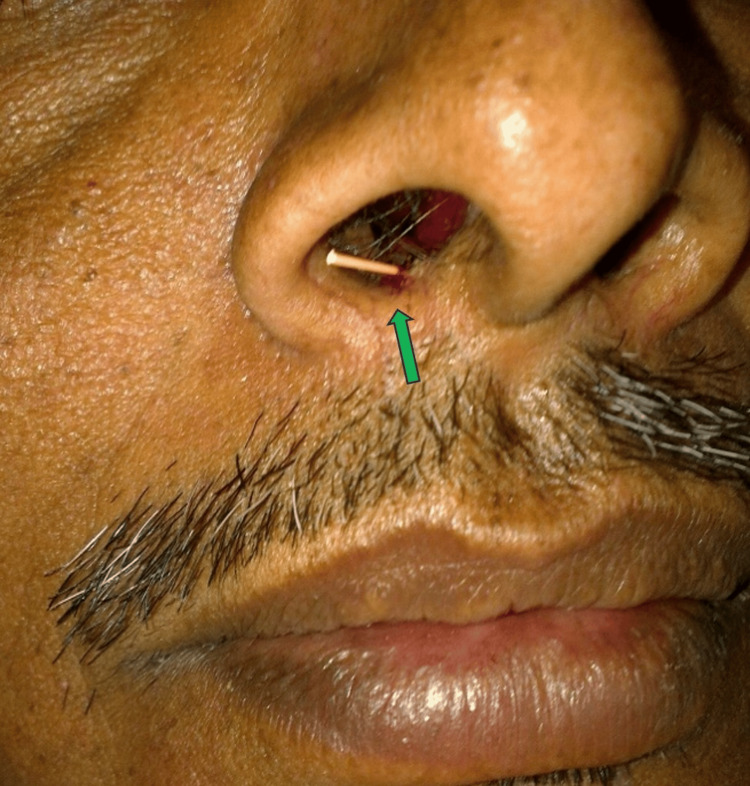
Gutta-percha cone inserted through the sinus tract (green arrow)

**Figure 4:**
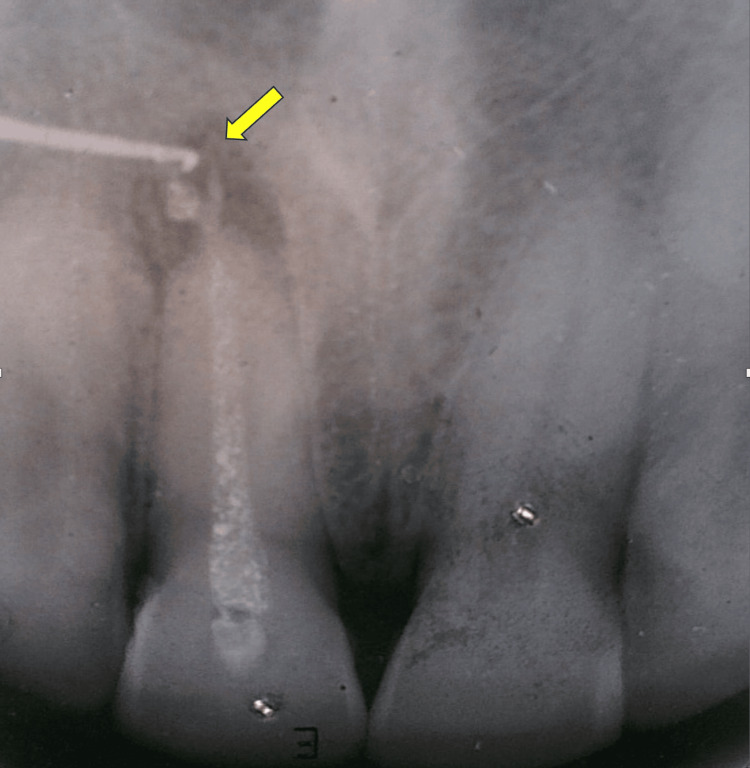
Gutta-percha tracing confirming the odontogenic origin of the periapical lesion (yellow arrow)

Initially, endodontic treatment was performed without local anesthesia, confirming the presence of necrotic pulp tissue in the non-vital tooth. Following access cavity preparation, the canal was instrumented using the ProTaper file system to an F3 master apical file, with a working length of 13.3 mm. Metapex was used as an intracanal medicament, and a calcium hydroxide dressing was placed to seal the canal. At the two-week follow-up, no suppuration or purulent discharge from the sinus tract was noted on digital compression. Because of the persistent infected periapical bony lesion, the dressing was replaced every two weeks. Radiographic evaluation showed only partial bone healing, although the patient had become completely asymptomatic. Therefore, periapical surgical curettage was performed to eliminate the infected granulation tissue. The canal was subsequently obturated with gutta-percha and indomethacin canal sealer, followed by placement of a temporary restoration.

Immediately afterward, an apicoectomy with root-end retrograde filling was performed. Before the procedure, the patient was instructed to rinse with a 1:2 dilution of Betadine gargle for three minutes. Local anesthesia was then administered, followed by elevation of a full-thickness mucoperiosteal flap and removal of the compromised tissue (Figure 5).

**Figure 5:**
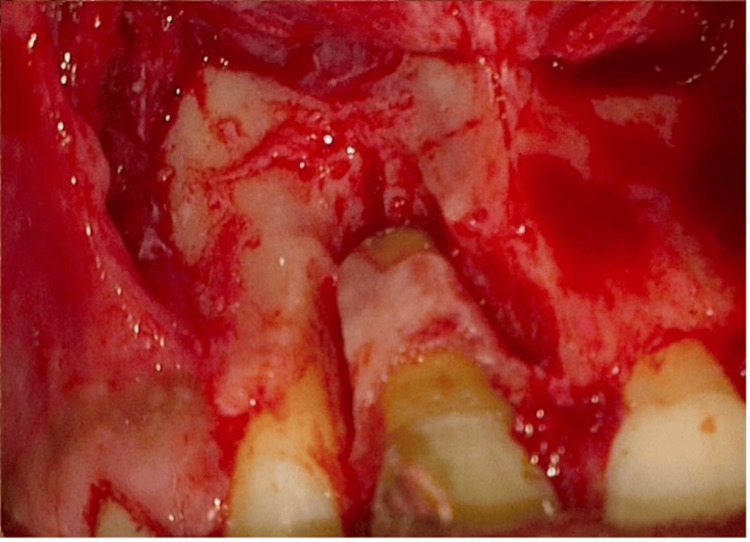
Periapical surgery and removal of granulation tissue

The patient's right maxillary central incisor became completely asymptomatic after 15 days. The granulation tissue was thoroughly curetted using a surgical bone curette. After complete removal of the granulation tissue, the surgical site was cleaned with ultrasonic instrumentation, followed by copious irrigation with saline.

Subsequently, the excess tissue tags were removed from the flap and the infected area. The flap was then repositioned and sutured using 3-0 (16 mm) reverse-cutting black silk sutures. Postoperative instructions were provided, and the patient was prescribed antibiotics and supportive medications (tablet amoxicillin/clavulanic acid 625 mg three times daily, tablet aceclofenac + paracetamol + serratiopeptidase twice daily, tablet pantoprazole 40 mg once daily before breakfast, and one probiotic capsule at bedtime). All medications were prescribed for seven days. The patient was recalled after seven days for follow-up. Thereafter, the patient was followed for 1-2 months with periodic radiographic evaluation. Complete healing was observed (Figure 6).

**Figure 6:**
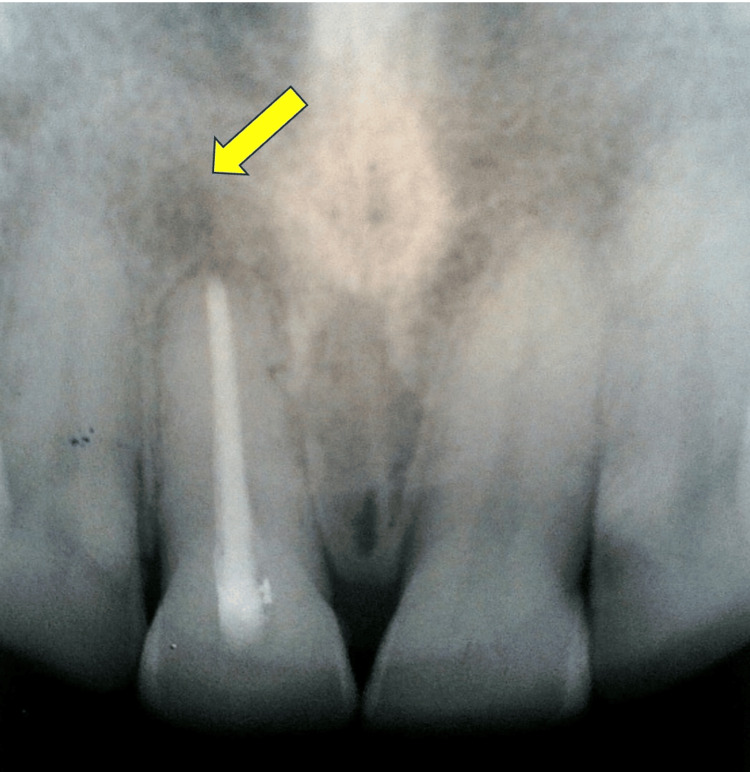
Follow-up intraoral periapical (IOPA) radiograph obtained after two months, showing complete healing of the periapical lesion (yellow arrow)

Hence, complete resolution of the lesion and nasal swelling was observed (Figure 7).

**Figure 7:**
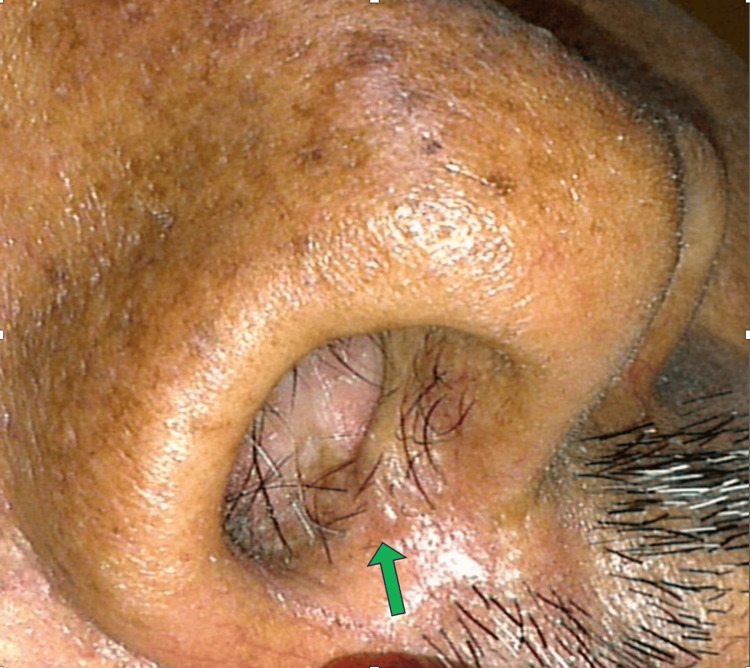
Complete resolution of the nasal sinus tract (green arrow)

## Discussion

When diagnosing a nasal sinus tract or facial fistula of dental origin, dentists usually evaluate for periodontal disease or dental caries, but should also consider the possibility of previous dental trauma [9]. Moreover, because the clinical presentation of a sinus tract may resemble that of other lesions, it can present a diagnostic challenge [10]. Despite advances in medical science, cases of misdiagnosed or improperly managed dental sinus tracts continue to be reported in the literature, as demonstrated in our case.

Most odontogenic infections result in the formation of an intraoral sinus tract. Gupta and Hasselgren, in their study of 29 cases, found that all odontogenic sinus tracts had intraoral openings [11], whereas Slutzky-Goldberg et al., in a cohort study of 108 odontogenic sinus tracts, reported only one case with a cutaneous sinus tract [12]. Miri et al. reported a frequency of 9.9% for odontogenic sinus tracts with extraoral openings [13]. Similarly, Sadeghi and Dibaei, in a study conducted in Iran, reported a prevalence of 14.9% for odontogenic sinus tracts with extraoral openings [14]. Collectively, these studies demonstrate the relatively rare occurrence of cutaneous sinus tracts. Our case involved an odontogenic sinus tract with a nasal opening, representing an unusual extraoral presentation.

Approximately 80% of reported odontogenic sinus tracts arise from mandibular teeth [10]. In contrast, the sinus tract in our case originated from a maxillary central incisor, making this presentation uncommon.

Our patient presented with black discoloration of the right maxillary central incisor due to pulp necrosis, accompanied by intermittent discharge from the nasal sinus tract and significant pain. Similarly, Belmehdi et al. reported an extraoral sinus tract near the nasolabial groove associated with discoloration of the right maxillary canine due to pulp necrosis and purulent discharge [10]. In our case, the IOPA showed a periapical radiolucency at the root apex of the right maxillary central incisor. Likewise, Belmehdi et al. reported apical radiolucency surrounding the apex of tooth 13 [10]. Hidoussi et al. reported a sinus tract beneath the chin in a 17-year-old boy following trauma to the lower lip [15]. The mandibular right central incisor was sensitive to percussion and demonstrated periapical radiolucency on radiographic examination. The case was diagnosed as a cutaneous sinus tract secondary to a chronic periapical abscess of the mandibular right central incisor [15]. These findings support the diagnosis of chronic apical periodontitis resulting from pulp necrosis, which led to the development of a cutaneous sinus tract involving the right nostril in our patient. Therefore, radiographic examination is valuable in diagnosing periapical lesions in such cases.

In our case, the sinus tract on the right side of the nostril was traced by inserting a gutta-percha cone toward the apex of the right maxillary central incisor, confirming its odontogenic origin. Similarly, Giménez-García et al. and Bouguezzi et al. traced sinus tracts using a gutta-percha cone [16,17].

Initially, endodontic treatment was performed without local anesthesia, confirming the presence of necrotic pulp tissue in the non-vital tooth. Metapex was used as an intracanal medicament, followed by placement of a calcium hydroxide dressing, similar to the protocols described by Cioffi et al. and Tidwell et al. [18,19]. At the two-week follow-up, no suppuration or purulent discharge from the sinus tract was observed. Although radiographic evaluation showed only partial bone healing, the patient had become completely asymptomatic. Therefore, periapical surgical curettage was performed, followed by obturation of the canal with gutta-percha and indomethacin canal sealer. A similar surgical approach was reported by Zemmouri and Chbicheb [20].

## Conclusions

This case involved the development of a periapical lesion in a maxillary central incisor that resulted in an extra-nasal sinus tract following accidental trauma. Following successful endodontic treatment and surgical curettage of the periapical lesion, the nasal lesion resolved completely. Clinicians should be aware of both odontogenic and non-odontogenic causes of lesions associated with sinus pathologies and should carefully evaluate the pathways of infection through thorough clinical and radiographic examination of both extraoral and intraoral structures. Adherence to appropriate diagnostic and treatment protocols is essential for successful patient management.
